# Comparative study of mitotic chromosomes in two blowflies, *Lucilia sericata* and *L. cluvia* (Diptera, Calliphoridae), by C- and G-like banding patterns and rRNA loci, and implications for karyotype evolution

**DOI:** 10.3897/CompCytogen.v9i1.8671

**Published:** 2015-03-31

**Authors:** Mónica G. Chirino, Luis F. Rossi, María J. Bressa, Juan P. Luaces, María S. Merani

**Affiliations:** 1 Instituto de Ecología, Genética y Evolución de Buenos Aires, Departamento de Ecología, Genética y Evolución, Facultad de Ciencias Exactas y Naturales, Universidad de Buenos Aires, Intendente Güiraldes 2160, C1428EHA Ciudad Autónoma de Buenos Aires, Argentina; 2 Laboratorio de Biología Cromosómica, Facultad de Medicina, Universidad de Buenos Aires, Paraguay 2155, C1121ABG Ciudad Autónoma de Buenos Aires, Argentina

**Keywords:** Blowflies, karyotype evolution, sex chromosomes, heterochromatin, G-like banding pattern, rDNA-FISH

## Abstract

The karyotypes of *Lucilia cluvia* (Walker, 1849) and *Lucilia sericata* (Meigen, 1826) from Argentina were characterized using conventional staining and the C- and G-like banding techniques. Besides, nucleolus organizer regions (NORs) were detected by fluorescent *in situ* hybridization (FISH) and silver staining technique. The chromosome complement of these species comprises five pairs of autosomes and a pair of sex chromosomes (XX/XY, female/male). The autosomes of both species have the same size and morphology, as well as C- and G-like banding patterns. The X and Y chromosomes of *Lucilia cluvia* are subtelocentric and easily identified due to their very small size. In *Lucilia sericata*, the X chromosome is metacentric and the largest of the complement, showing a secondary constriction in its short arm, whereas the Y is submetacentric and smaller than the X. The C-banding patterns reflect differences in chromatin structure and composition between the subtelocentric X and Y chromosomes of *Lucilia cluvia* and the biarmed sex chromosomes of *Lucilia sericata*. These differences in the sex chromosomes may be due to distinct amounts of constitutive heterochromatin. In *Lucilia cluvia*, the NORs are placed at one end of the long-X and of the long-Y chromosome arms, whereas one of the NORs is disposed in the secondary constriction of the short-X chromosome arm and the other on the long-Y chromosome arm in *Lucilia sericata*. Although the G-like banding technique does not yield G-bands like those in mammalian chromosomes, it shows a high degree chromosomal homology in both species because each pair of autosomes was correctly paired. This chromosome similarity suggests the absence of autosomal rearrangements during karyotype evolution in the two species studied.

## Introduction

The dipteran family Calliphoridae (blowflies) includes several common synanthropic forms, most of which have saprophagous habits. Some blowflies are considered a serious public health problem since certain species can cause myiasis in humans and domestic animals. Other blowflies are of great medical, veterinary, and forensic importance (Hanski and Kuusela 1980, Kuusela and Hanski 1982, Greenberg 1991, Martínez Sánchez et al. 2000, Centeno et al. 2002, 2004, Agrawal et al. 2010, Davydov 2011). Calliphorids are recognized as the first wave of faunal succession on human cadavers (Nuorteva 1977, Smith 1986) and, therefore, are the primary and most accurate indicators of the time of death (Centeno et al. 2002, Ames and Turner 2003). The larvae of certain facultative parasites are used in maggot therapy to treat infected chronic wounds in humans and vertebrates by allowing the removal of necrotic tissue, which induces the formation of granular tissue and the growth of healthy skin (Sherman 2002, Horobin et al. 2005, Parnés and Lagan 2007, Davydov 2011).

Within Calliphoridae, *Lucilia cluvia* (Walker, 1849) and *Lucilia sericata* (Meigen, 1826) are among the most abundant exploiters of carcasses and faeces as food sources, oviposition sites, and sites for larval development, being their biological development very important in the field of forensic science. *Lucilia sericata* is one of the first insects to arrive at a corpse and their immature flies are used to estimate the minimum portion of the *post-mortem* interval, known as *PMI* (Hanski and Kuusela 1980, Kuusela and Hanski 1982, Martínez Sánchez et al. 2000, Centeno et al. 2002, Rueda et al. 2010). The larvae of *Lucilia sericata*, *Lucilia cluvia*, and *Lucilia illustris* (Meigen, 1826) are the most suitable and effective facultative parasites used in human wound treatment of injuries that conventional treatments fail to heal (Sherman and Wyle 1996, Bani-Ardalani 2005, Zapata et al. 2008, Rueda et al. 2010, Thyssen et al. 2013). Uncertain or incorrect taxonomic identification of maggot and/or imaginal stages could have unpredictable consequences for the implementation of larvae of different blowfly species in the larval therapy. Therefore, cytogenetic studies have significant value because they allow differentiating between related species that are cryptic and/or morphologically similar, particularly in their larval stages, and provide information and useful diagnostic characters at the species level.

In Calliphoridae, cytogenetic data are scarce and refer almost exclusively to the karyotype, C-banding and/or the C value of a very few species (Boyes and van Brink 1965, Boyes and Shewell 1975, Bedo 1980, 1991, Parise-Maltempi and Avancini 2001, El-Bassiony 2006, Ullerich and Schöttke 2006). This family shows a remarkably uniform karyotype (2n = 12), generally comprising five pairs of large euchromatic autosomes and a pair of heteromorphic sex chromosomes. Previous reports have revealed a great deal of similarity among autosomes and variation in size and morphology of the sex chromosomes from species to species (Boyes and Shewell 1975, Bedo 1980, Ullerich and Schöttke 2006, Agrawal et al. 2010). Thirty-two species of *Lucilia* were taxonomically described (Stevens and Wall 1996, Whitworth 2010, 2014), but only seven species were studied. These are *Lucilia sericata* from Africa and Germany, *Lucilia illustris* and *Lucilia caesar* (Linnaeus, 1758) from Germany and Japan, *Lucilia porphyrina* Walker, 1856 and *Lucilia ampullacea* Villeneuve, 1922 from Japan, *Lucilia eximia* (Wiedemann, 1819) from Brazil, and *Lucilia cuprina* (Wiedemann, 1830) from Australia (Boyes and Shewell 1975, El-Bassiony 2006, Ullerich and Schöttke 2006, Agrawal et al. 2010). In *Lucilia* species, the autosomes are less variable and very seldom appear to be heterochromatic as compared to sex chromosomes, which show a considerable interspecific variation in size and shape. *Lucilia illustris* has a relatively long X chromosome whereas *Lucilia ampullacea* and *Lucilia caesar* have short heteromorphic sex chromosomes (Boyes and Shewell 1975, Bedo 1991, Ullerich and Schöttke 2006, Agrawal et al. 2010).

In the present work, we examined and compared the karyotype of *Lucilia cluvia* and *Lucilia sericata* from Argentina. We analysed the constitutive heterochromatin content and distribution, and identified the nucleolus organizer regions (NORs) in female and male mitotic chromosomes of these species by means C-banding and fluorescent *in situ* hybridization (FISH) with 18S rDNA heterologous probes, respectively. In order to confirm the accurate detection of ribosomal genes location, we also applied the silver impregnation for staining NORs on mitotic chromosomes that were transcriptionally active during the preceding interphase. We also identify the chromosome pairs in both species by means of G-like banding. Finally, we discuss our cytogenetic results and compare them with those previously described.

## Materials and methods

### Fly sources

*Lucilia cluvia* and *Lucilia sericata* occurring in grasslands, shrubs and forest habitats in open areas near Buenos Aires City (34°36'14"S and 58°22'54"W), Argentina, were collected using beef meat as baits between January and May 2014. For mitotic analysis, chromosome bandings and fluorescent *in situ* hybridization (FISH) technique, 7 females of *Lucilia cluvia* and 20 females of *Lucilia sericata* were collected. Flies were identified using Mariluis and Schnack key (2002). After identification, the females were transferred into a cage for oviposition at 22 ± 2 °C, and 60 ± 5% relative humidity. The rearing cages were supervised daily and flies were allowed to develop into third-instar (L3) larvae. The number of egg-clusters oviposited by each female was ranked between 200–250 eggs.

### Chromosome preparations

Mitotic chromosomes were obtained from the neural ganglia of L3 larvae. At least 100 females and males (20–30 larvae for each sample) of each species were analysed. Larvae were injected with 0.01 ml of colchicine (0.1 mg/ml) and dissected under a stereomicroscope after 45 min treatment. Brains were dissected using fine forceps, dispersed in 3 ml of KCl 0.075 M for 15 min at 25 °C, centrifuged at 600 rpm for 10 min, and fixed in methanol:acetic acid (3:1) overnight. The cell suspension was dropped onto clean slides using air-drying technique (Rothfels and Siminovitch 1957, Chirino et al. 2014). For karyotype analysis and chromosome length measurements, chromosome preparations were made from brains of untreated larvae with colchicine.

### Chromosome bandings

C-banding was performed according to Sumner (1972) with modifications: slides were treated with 0.2 N HCl for 20 min at room temperature, 5% saturated solution of Ba(OH)_2_ at 50 °C for 1–2 min and 2X SSC at 60 °C for 60 min; slides were then stained with 3% Giemsa solution at pH 6.8 for 20–30 min. G-like banding was performed following the method of Wang and Fedoroff (1972) with modifications: within 96 h after air drying, slides were incubated in phosphate buffered saline (PBS) for 10 min and in 0.1%, 0.05% and 0.025% trypsin for 1–6 min; slides were then air-dried and stained with 1% Giemsa solution at pH 6.8 for 5–10 min.

Detection of the nucleolus organizer regions (NORs) on mitotic chromosomes was done following the silver staining method of Howell and Black (1980) with slight modifications. Onto the slides, 30 μl of 1% aqueous gelatine solution with 0.5% formic acid and 20 μl drops of silver nitrate at 50% were dropped. The slides were covered with coverglass and incubated at 45 °C for 2–10 min until the silver staining mixture became yellowish. The slides were washed with distilled water, air-dried, and examined immediately under microscope.

### Fluorescence in situ hybridization

Unlabelled 18S ribosomal DNA (rDNA) probes, derived from genomic DNA of the true bug, *Dysdercus albofasciatus* Berg, 1878 (Heteroptera: Pyrrhocoridae), were obtained and labelled with biotin 14-dATP by nick translation using a BioNick Labeling System (Invitrogen, Life Technologies Inc., San Diego, CA, USA) as described in Fuková et al. (2005) and Bressa et al. (2009). FISH with a biotinylated 18S rDNA probe was performed following the procedure described in Bressa et al. (2009). Hybridization signals were detected with Cy3-conjugated streptavidin (Jackson ImmunoRes. Labs. Inc., West Grove, PA, USA).

### Microscopy, photographs and image processing

Fifty mitotic metaphases of females and males per individual of *Lucilia cluvia* and *Lucilia sericata* were analysed to determine the karyotype of each species. Ten metaphases of each species (*Lucilia cluvia* and *Lucilia sericata*) were used to perform each species idiogram. Lengths of chromosomes were calculated and expressed as percentage of the female haploid set. Measurements were also performed on five banded karyotypes of each species to avoid errors in chromosome identification. At least 40 G-like banded, 20 C-banded, 20 rDNA-FISH, and 20 Ag-NOR cells for each gender and species were examined and photographed. Idiograms illustrating the G-like banding patterns were obtained.

## Results

### Chromosome complement

The female and male karyotypes and C-banding pattern of *Lucilia sericata* were already reported (El-Bassiony 2006, Ullerich and Schöttke 2006). However, we examined in detail different karyotype features of this species, such as chromosome banding patterns, percentage of heterochromatin and number and location of NORs. As a result, we made a comparison study of the mitotic karyotypes of both *Lucilia* species.

**Figure 1.**
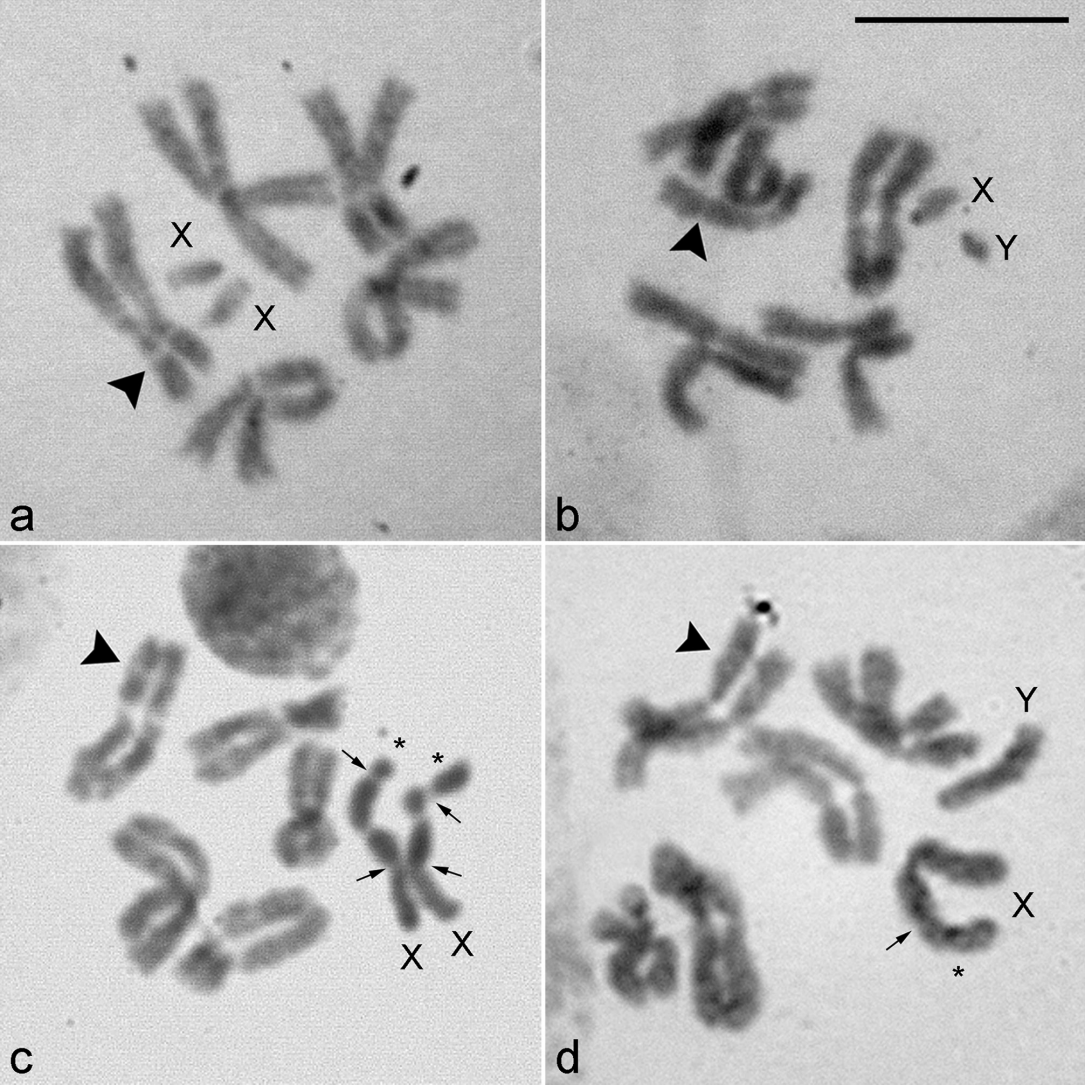
Female and male karyotypes of *Lucilia cluvia* (**a–b**) and *Lucilia sericata* (**c–d**), 2n = 10 + XX/XY, stained with 3% Giemsa. X, Y = sex chromosomes. Arrowheads show the secondary constriction in chromosome 2. Arrows show the secondary constriction in the X chromosomes. Asterisks indicate the satellite. Bar = 10 μm.

The diploid chromosome complements of *Lucilia cluvia* and *Lucilia sericata* are 2n = 12, consisting of five large biarmed autosomal pairs and one sex chromosome pair (XX/XY, female/male; Fig. 1). In both species, *Lucilia cluvia* (Fig. 1a–b) and *Lucilia sericata* (Fig. 1c–d), the autosomes show a very close somatic pairing, the sex chromosomes tend to not pair with each other or stay unpaired, and all the chromosomes are similar in morphology and size, except for the sex chromosomes (Table 1). In female and male somatic metaphases of *Lucilia cluvia* (Fig. 1a–b) and *Lucilia sericata* (Fig. 1c–d), pairs 1, 2, 4 and 5 are metacentric chromosomes, whereas pair 3 comprises two submetacentric chromosomes. Pair 2 possesses a secondary constriction in the short arm.

**Table 1.** Comparison of the relative lengths of chromosomes of *Lucilia cluvia* and *Lucilia sericata* in % of the haploid set (mean ± SE).

Chromosome pair	TL ^†^	Short arm (p)	Long arm (q)	AI (classification) ^‡^
*Lucilia cluvia*				
1	23.24 ± 1.95	10.11 ± 0.67	12.48 ± 0.91	0.81 ± 0.05 (M)
2	18.63 ± 1.10	7.97 ± 0.66	9.22 ± 0.69	0.87 ± 0.09 (M)
3	18.35 ± 0.55	5.93 ± 0.53	10.93 ± 0.38	0.54 ± 0.06 (SM)
4	17.43 ± 2.07	6.16 ± 0.50	10.28 ± 1.91	0.62 ± 0.14 (M)
5	16.51 ± 0.44	7.36 ± 0.24	8.47 ± 0.28	0.87 ± 0.05 (M)
X	5.84 ± 0.86	1.26 ± 0.21	3.82 ± 0.74	0.35 ± 0.12 (ST)
Y	4.77 ± 0.28	1.07 ± 0.18	3.46 ± 0.26	0.31 ± 0.07 (ST)
*Lucilia sericata*				
1	19.44 ± 0.24	8.71 ± 0.29	9.59 ± 0.40	0.91 ± 0.07 (M)
2	17.55 ± 0.89	7.18 ± 1.04	9.13 ± 0.82	0.80 ± 0.19 (M)
3	15.18 ± 0.34	4.82 ± 0.78	8.80 ± 0.49	0.55 ± 0.12 (SM)
4	14.77 ± 0.32	5.34 ± 0.60	8.60 ± 0.48	0.58 ± 0.09 (M)
5	11.95 ± 0.40	5.67 ± 0.28	6.64 ± 0.37	0.78 ± 0.09 (M)
X	19.97 ± 1.35	8.75 ± 0.31	10.77 ± 0.97	0.82 ± 0.05 (M)
Y	13.70 ± 2.20	4.75 ± 1.39	8.15 ± 0.50	0.58 ± 0.14 (SM)

† TL = total length.

‡ AI = arm index; M = metacentric; SM = submetacentric; ST = subtelocentric.

In mitotic metaphases of *Lucilia cluvia*, the X and Y chromosomes are subtelocentric and are easily identified among the remaining five pairs of autosomes due to their very small size (Fig. 1a–b; Table 1), being the Y chromosome slightly smaller than the X chromosome (Fig. 1b). On the other hand, the X chromosome of *Lucilia sericata* is metacentric and the longest of the diploid set, representing 20.0% of the haploid set. The Y sex chromosome is submetacentric, smaller than the X chromosome and represents 13.7% of the set (Fig. 1c–d). In female of this species, both X chromosomes present a secondary constriction in their short arms and a satellite at terminal position (Fig. 1c; Table 1). Both X chromosomes can be distinguished due to the different size of their satellites, leading to a significant increase in their size (Table 1).

### Chromosome bandings

**Figure 2.**
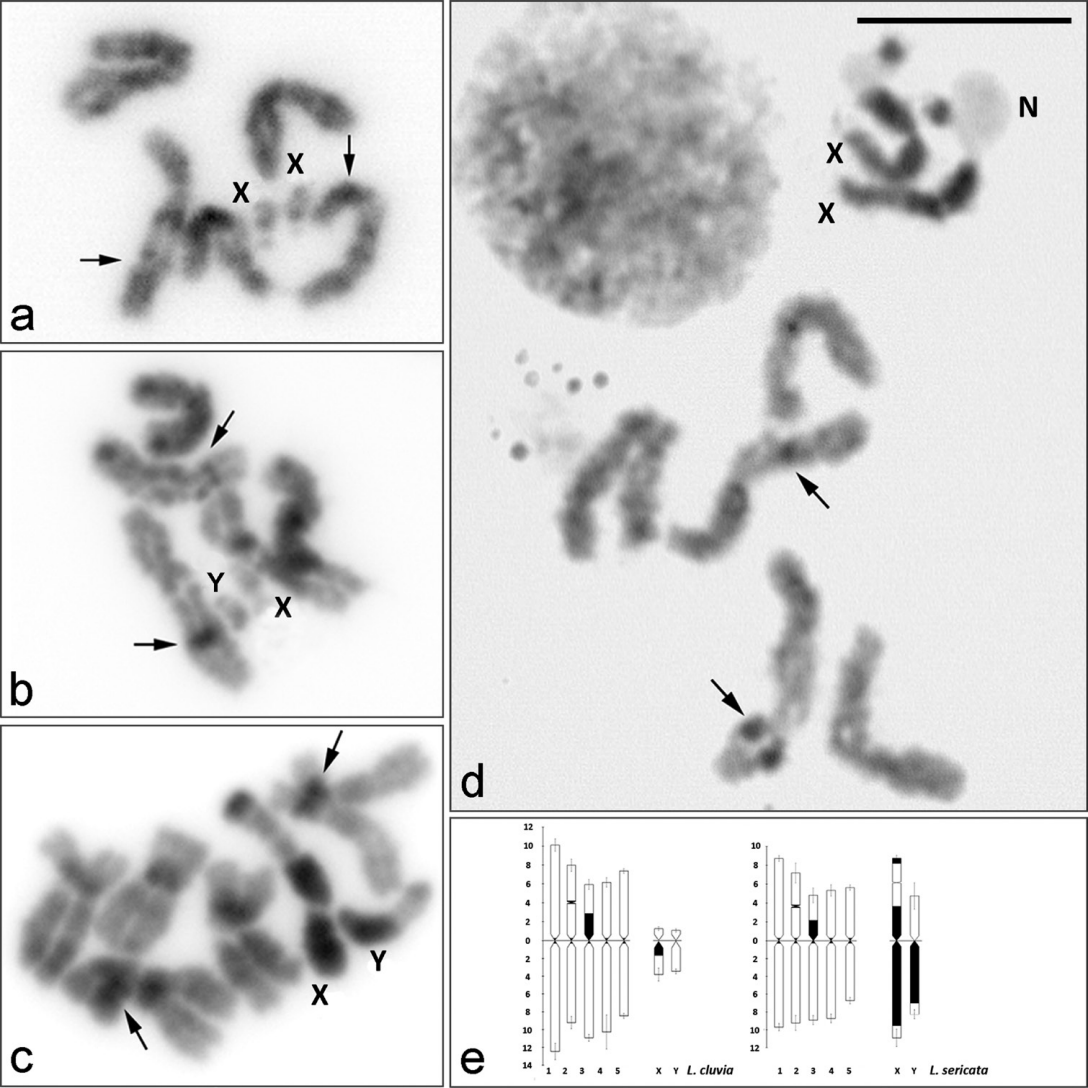
C-banding on female and male mitotic chromosomes of *Lucilia cluvia* (**a–b**) and *Lucilia sericata* (**c–d**), stained with 3% Giemsa, and C-banded idiograms of autosomes and sex chromosomes of *Lucilia cluvia* and *Lucilia sericata* (**e**). X, Y = sex chromosomes. N = nucleolus. Arrows indicate C-positive heterochromatin bands at the secondary constriction in chromosome 2 and at interstitial position in chromosome 3. Bar = 10 μm.

The C-banding pattern of autosomes is mainly limited to a single narrow band at the centromeric region in each of five pairs in both species of *Lucilia* (Fig. 2). Besides, two interstitial bands are observed on each short arm of chromosomes 2 and 3. In the former, the C-band is associated with a secondary constriction (Fig. 2). Both species show differences in the C-banding pattern in the X and Y sex chromosomes. In *Lucilia cluvia*, the X chromosome has a small C-positive band located in the proximal pericentromeric region of its long arm, whereas the Y chromosome is euchromatic (Fig. 2a–b, e). In *Lucilia sericata*, the satellite and the proximal region of the short X-chromosome arm are heterochromatic (Fig. 2c–e), except the secondary constriction where a single nucleolus is located and observed as a negative heteropyknotic body (Fig. 2d–e). The long X-chromosome and the long Y-chromosome arms are almost heterochromatic, except the distal regions (Fig. 2c–e). From these results, we found significant differences in the content and distribution of constitutive heterochromatin in *Lucilia cluvia* and *Lucilia sericata*. In the former, the constitutive heterochromatin is mainly located on the chromosome X (3.1% of the total chromosome length) and the autosomal pair 3 (1.8%). In the latter, most of constitutive heterochromatin is found on the X (15.4% of its total length) and to a lesser extent on the Y (8.4%) and pair 3 (2.4%).

**Figure 3.**
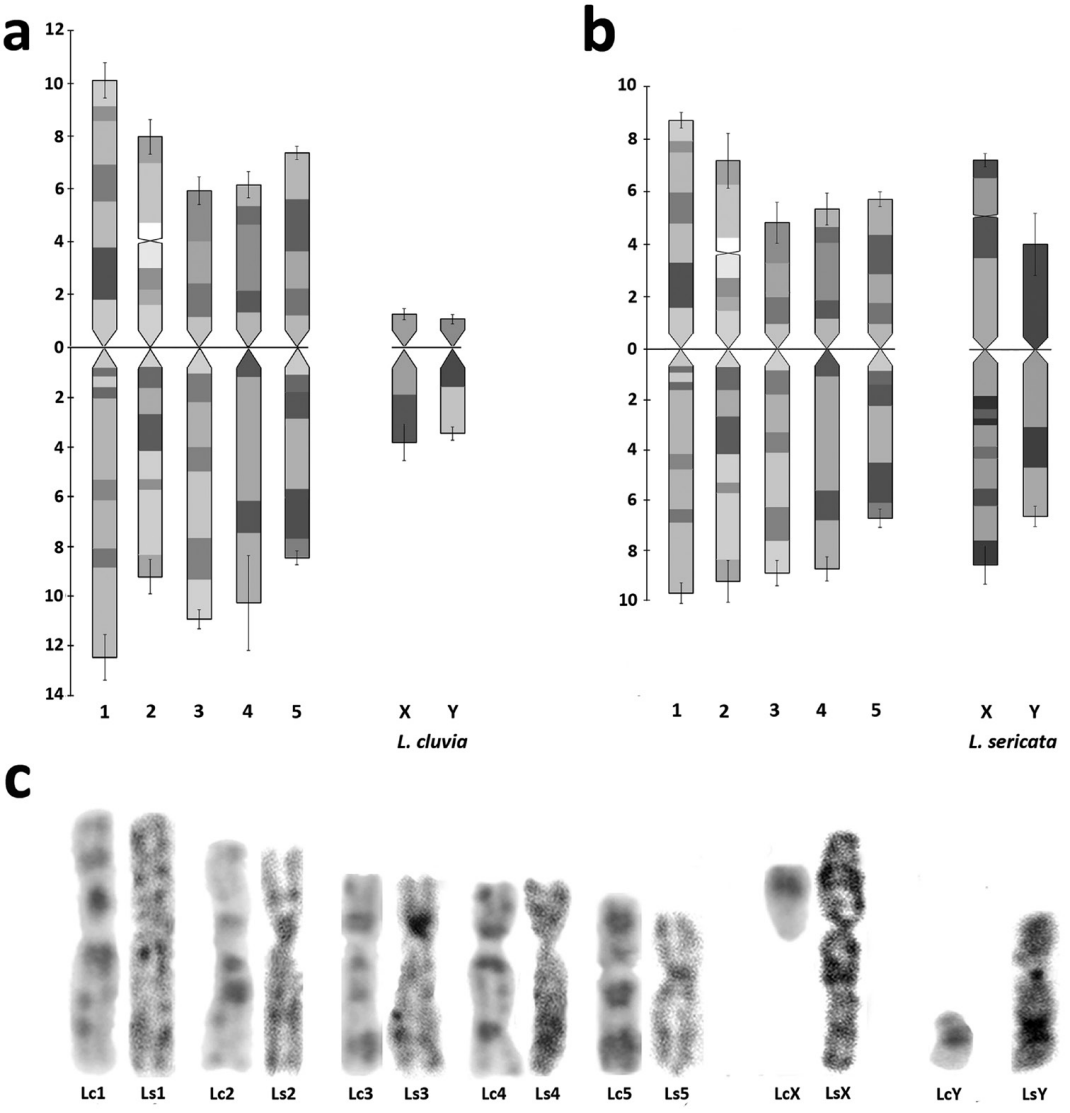
Pattern of G-like bands in the ideogram of *Lucilia cluvia* (**a**) and *Lucilia sericata* (**b**), and G-like banding homology between chromosomes of *Lucilia cluvia* (Lc) and *Lucilia sericata* (Ls) (**c**) revealing a high degree of conservation in G-like banding patterns between homologous chromosomes.

The homology between the karyotypes of *Lucilia cluvia* and *Lucilia sericata* is illustrated in Figure 3. In all mitotic chromosome preparations, G-like bands are very evident and always present on homologous chromosomes from *Lucilia cluvia* and *Lucilia sericata*. These bands dispersed along the chromosomes are useful for idiogram reconstruction (Fig. 3a–b). The distribution of G-like banding pattern was homologated in both species of *Lucilia*, and the chromosomal homology in their karyotypes was observed since each pair of autosomes was correctly paired (Fig. 3a, b). The distribution of G-like bands in the X sex chromosomes of *Lucilia cluvia* was coincident to that observed in the short arm of the X chromosomes of *Lucilia sericata* (Fig. 3c).

### Localization of rDNA by FISH and Ag-NOR banding

**Figure 4.**
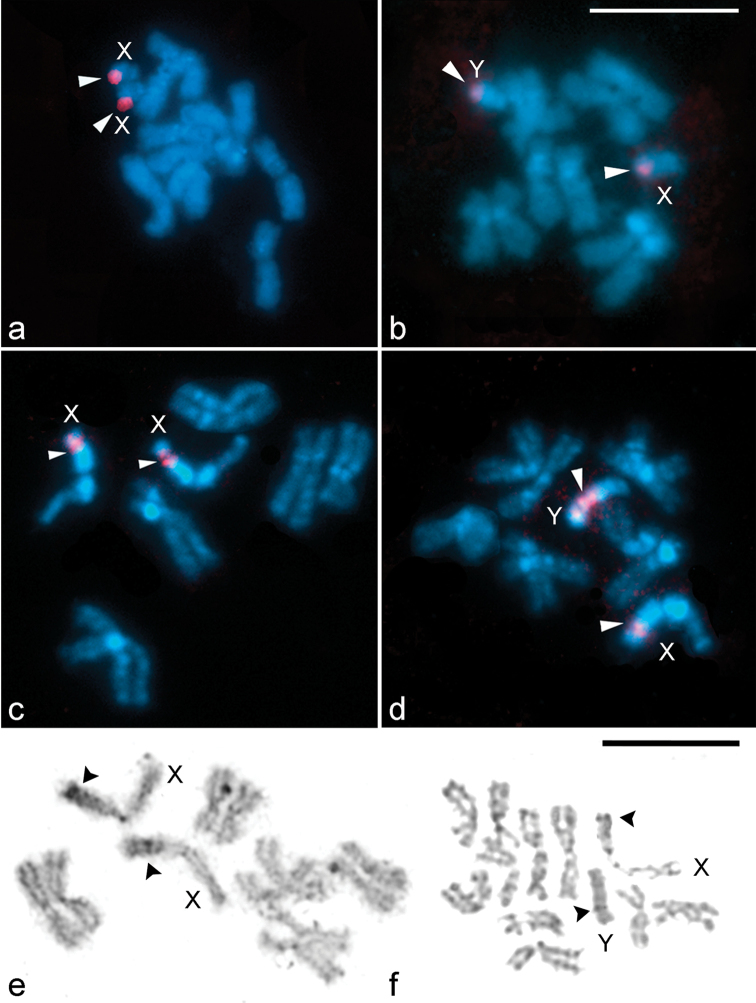
Location of rDNA genes and Ag-NOR sites on female and male mitotic chromosomes of *Lucilia cluvia* (**a–b**) and *Lucilia sericata* (**c–d**, **e–f**) using FISH with 18S rDNA probes (red signals, arrowheads) and silver impregnation technique. Chromosomes were counterstained with DAPI (blue). X, Y = sex chromosomes. Arrowheads indicate hybridization signals (**a–d**) and Ag-NOR sites (**e–f**) in both sex chromosomes. Bar = 10 μm.

In preparations of mitotic metaphases from both sexes of *Lucilia cluvia* and *Lucilia sericata*, FISH experiments with the 18S rDNA probe show two clusters of rDNA genes, one of them located on the X chromosome and the other on the Y chromosome (Fig. 4). In females of *Lucilia cluvia*, a single cluster of hybridization signals is regularly observed at terminal region of long arm of each X chromosome (Fig. 4a). In males, the hybridization signals are observed both at the end of the long-X chromosome arm as at the terminal position on the long-Y chromosome arm (Fig. 4b). In females of *Lucilia sericata*, the rDNA probe displays strong hybridization signals in the secondary constriction of the short arm of both X chromosomes (Fig. 4c). In male metaphase complements, the hybridization signals are clustered in the secondary constriction of the short X-chromosome arm and in the proximal region of the large arm of the Y chromosome (Fig. 4d). In most male metaphases of *Lucilia sericata*, the intensity of the hybridization signals differs between the two sex chromosomes, with the Y chromosome showing stronger and larger clusters of signals (Fig. 4d).

A single nucleolus is present on both X and Y sex chromosomes of *Lucilia sericata* (Fig. 4e–f). At female mitotic metaphases, positive Ag-NORs are observed in the secondary constriction of the short arm of both X chromosomes (Fig. 4e). Male mitotic metaphases show positive Ag-NORs in the secondary constriction of the short X-chromosome arm and in the large arm of the Y chromosome (Fig. 4f).

## Discussion

The karyotypes of the blowflies *Lucilia cluvia* and *Lucilia sericata* examined in the present study agree fairly well with the earlier findings known from other *Lucilia* species and members of Calliphoridae. The species of this family show remarkable karyotypic uniformity with 2n = 12, comprising five pairs of large or medium-sized meta/submetacentric autosomes and a heteromorphic XX/XY sex chromosome pair (female/male) (Boyes and Shewell 1975, Azeredo-Espin and Pavan 1983, Parise-Maltempi and Avancini 2001, Ullerich and Shöttke 2006, Agrawal et al. 2010, Holecová et al. 2012).

Our analysis of mitotic chromosomes based on conventional staining and C- and G-like bandings revealed a homology in the five pairs of autosomes and a noticeable sex chromosome variation with respect to morphology, size and heterochromatin content in metaphase karyotypes of *Lucilia cluvia* and *Lucilia sericata*. The autosomes of Calliphoridae reveal a great deal of stability as compared to the sex chromosomes, which show variation in shape and size from one species to another (Boyes and Shewell 1975, Azeredo-Espin and Pavan 1983, Parise-Maltempi and Avancini 2001, Ullerich and Shöttke 2006, Agrawal et al. 2010, Holecová et al. 2012). Moreover, the autosomes in *Lucilia cluvia* and *Lucilia sericata* exhibited a very close somatic pairing (i.e. side-by-side pairing), a characteristic feature of chromosome complement of all the dipterans where the homologous chromosomes tend to lie next to one another. Consequently, the diploid complements give the appearance of a haploid set (Agrawal et al. 2010). Nonetheless, the sex chromosomes XX in females and XY in males did not show such intimate somatic pairing and tended to lie separately (Boyes and Shewell 1975, Ullerich and Shöttke 2006, Agrawal et al. 2010, Holecová et al. 2012, this study).

In *Lucilia cluvia* and *Lucilia sericata*, some characteristics of the karyotype and C-banding described herein resemble those previously reported and those of closely related species (Bedo 1980, El-Bassiony 2006, Ullerich and Shöttke 2006): i) the autosome pairs of both species decrease gradually in size and present small centromeric C-positive bands, and ii) the X chromosome of *Lucilia sericata*, which is larger than autosome pair 1, has in its short arm a satellite and a subterminal secondary constriction where a single nucleolus is located. However, we observed the presence of interstitial C-positive heterochromatic bands in autosome pairs 2 and 3, and significant differences in morphology and C-banding pattern of the Y chromosome. In the Argentine population of *Lucilia sericata*, the short arm of the Y submetacentric chromosome was found to be completely euchromatic and only the long Y-chromosome arm was mainly C-positive, whereas the Y telocentric chromosome from the African population is entirely C-banded (Ullerich and Shöttke 2006). A clear distinction between the populations from Argentina and Africa could be established due to the amount and distribution of constitutive heterochromatin in autosomes and in the Y sex chromosome. The data presented herein reveal a substantial polytypic variation in *Lucilia sericata* and indicate that this chromosome polytypism might be due to the difference in gain of constitutive heterochromatin in the genome of both geographically isolated populations. Further studies are needed to clarify the relationship between heterochromatin content and the geographical, ecological or environmental characteristics of the species under study.

*Lucilia cluvia* and *Lucilia sericata* showed a high degree of similarity since homology of each autosome pair was established throughout G-like banding patterns, suggesting the absence of chromosome rearrangements in autosomes of both species and maybe within the genus *Lucilia* during karyotype evolution. Hence, the autosome pairs of *Lucilia cluvia* and *Lucilia sericata* were homologated by size and morphology, as well as by C- and G-like banding patterns. Considering the strong similarity of autosomes in Diptera calyptrate muscoid (Foster et al. 1980) together with the results here presented, we may infer that the autosomes of Calliphoridae retain a high degree of structural integrity and morphological stability.

The most remarkable interspecific dissimilarity of the *Lucilia* species herein studied is related to the X and Y sex chromosomes that show considerable variability in size, shape, and chromosome organization. Our results show that in *Lucilia cluvia*, the X and Y chromosomes are subtelocentric and the smallest of the complement, with the proximal pericentromeric region of the long-X chromosome arm heterochromatic and the Y chromosome euchromatic. In contrast, the X chromosome is metacentric and the longest of the complement and the Y chromosome is a medium-sized submetacentric in *Lucilia sericata*, being both of them mainly heterochromatic. In closely related species, the genome-size differences may be wholly explained by differential amounts of non-coding DNA (i.e. transposable elements, satellite DNAs, simple sequence repeats) (Gregory and Hebert 1999, Graur and Li 2000, Petrov 2001), and caused by diverse mechanisms such as duplications, deletions, genome mutations, activity of transposable elements, and amplification, accumulation or elimination of heterochromatin (Petrov 2001). Most segments of constitutive heterochromatin on eukaryotic chromosomes contain high concentrations of highly repeated (satellite) DNA and vary in composition and in length within and among species (Sumner 2003). Several dipteran species present different degrees of heterochromatinization in their sex chromosomes (Boyes and Shewell 1975, Bedo 1980, 1991, Baimai 1998, Parise-Maltempi and Avancini 2000, 2001, 2007, El-Bassiony 2006, Ullerich and Shöttke 2006, Agrawal et al. 2010, Holecová et al. 2012). Boyes and van Brink (1965) showed a tendency for the X chromosome, and to a lesser degree the Y, to accumulate heterochromatin in several subfamilies of calyptrate Diptera. Based on our findings, the interspecific size differences in the sex chromosomes of these *Lucilia* species could be due to differential amounts of constitutive heterochromatin, resulting from the amplification of pre-existing heterochromatin and/or the loss and/or gain of new heterochromatin.

In the present study, FISH experiments using 18S rDNA heterologous probes revealed two rDNA clusters in *Lucilia cluvia* and *Lucilia sericata*, one located in the X and the other one in the Y chromosome. The accurate detection of ribosomal genes on both sex chromosomes in *Lucilia sericata* was confirmed by means of silver impregnation. The determination of the number and location of the NORs makes them essential cytological markers for the study of karyotype structure and chromosome evolution since the rDNA genes are noticeably conserved among dipteran species. In most species studied, the NORs are located in the sex chromosomes (Bedo and Howells 1987, Bedo 1992, Willhoeft and Franz 1996, Willhoeft 1997). Considering the previous cytogenetic reports together with the mitotic karyotype, C-banding pattern and rDNA-FISH results here presented, we propose that in *Lucilia sericata* the X and Y sex chromosomes accumulated a sufficient number of repetitive DNA sequences, leading to an increase in chromosome size.

Among these blowflies, there are some cryptic or isomorphic species, which cause great taxonomic problems because of their similarity in external morphology of maggot and/or imago stages. The results presented here showed that the C- and G-like bands, and rDNA loci may be considered as essential cytological markers to compare karyotypes of phylogenetically related species and, also, of sibling species. Besides, the use of these approaches may also contribute to the analysis of changes in karyotype related to the evolutionary process and to a better understanding of taxonomic relationships.
